# Supplementary material for: Whole Genome Sequence Analysis of Salmonella Typhi Isolated in Thailand before and after the Introduction of a National Immunization Program
Source: PLoS Negl Trop Dis. 2017 Jan 6;11(1):e0005274. doi: 10.1371/journal.pntd.0005274 (PMC5245908; doi:10.1371/journal.pntd.0005274)
Supplement: S1 Table — (DOCX) [file pntd.0005274.s001.docx]

**S1 Table: Isolate and sequencing details**

| Sample Accession | Raw data accession | Sanger name | Strain name | Strain ID | Contributor name | Isolation location | Isolation date | Genotype | Read depth (-fold) | Resistance Genes | Plasmid replicons |
| --- | --- | --- | --- | --- | --- | --- | --- | --- | --- | --- | --- |
| ERS447209 | ERR586717 | 13244_5#20 | Ty002 | AFA-11489-T1 | AFRIMS | Bangkok | November, 1973 | 3.2.1 | 93.3 | - | - |
| ERS447208 | ERR586795 | 13244_6#5 | Ty003 | AFA-11496-T1 | AFRIMS | Bangkok | November, 1973 | 3.2.1 | 104.7 | - | - |
| ERS447207 | ERR586794 | 13244_6#4 | Ty004 | AFA-11686-T1 | AFRIMS | Bangkok | November, 1973 | 2.0.0 | 101.2 | - | pHCM2, X1 |
| ERS447206 | ERR586793 | 13244_6#3 | Ty005 | AFA-11689-T1 | AFRIMS | Bangkok | November, 1973 | 4.1.0 | 110.4 | - | - |
| ERS447205 | ERR586792 | 13244_6#2 | Ty006 | AFA-12717-T1 | AFRIMS | Bangkok | November, 1973 | 3.2.1 | 105.3 | - | - |
| ERS447204 | ERR586791 | 13244_6#1 | Ty007 | AFA-12719-T1 | AFRIMS | Bangkok | November, 1973 | 4.1.0 | 111.5 | - | - |
| ERS447203 | ERR586716 | 13244_5#19 | Ty008 | AFA-12722-T1 | AFRIMS | Bangkok | November, 1973 | 3.2.1 | 96.9 | - | - |
| ERS447211 | ERR586718 | 13244_5#21 | Ty009 | AFA-12727-T1 | AFRIMS | Bangkok | November, 1973 | 2.4.0 | 92.1 | - | - |
| ERS447212 | ERR586719 | 13244_5#22 | Ty011 | AFA-12731-T1 | AFRIMS | Bangkok | November, 1973 | 3.2.1 | 92.6 | - | - |
| ERS447213 | ERR586796 | 13244_6#6 | Ty012 | AFA-12733-T1 | AFRIMS | Bangkok | November, 1973 | 3.2.1 | 102.0 | - | - |
| ERS447214 | ERR586797 | 13244_6#7 | Ty013 | AFA-12738-T1 | AFRIMS | Bangkok | November, 1973 | 4.1.0 | 107.0 | - | - |
| ERS447215 | ERR586798 | 13244_6#8 | Ty014 | AFA-11771-T1 | AFRIMS | Nonthaburi | February, 1981 | 2.2.0 | 112.0 | - | - |
| ERS447217 | ERR586799 | 13244_6#9 | Ty016 | AFA-11775-T1 | AFRIMS | Nonthaburi | February, 1981 | 2.3.4 | 106.0 | - | - |
| ERS447219 | ERR586800 | 13244_6#10 | Ty018 | AFA-11768-T1 | AFRIMS | Nonthaburi | March ,1981 | 2.2.0 | 93.6 | - | - |
| ERS447220 | ERR586720 | 13244_5#24 | Ty020 | AFA-12439-T1 | AFRIMS | Bangkok | June, 1984 | 2.4.0 | 94.7 | - | - |
| ERS447221 | ERR586801 | 13244_6#11 | Ty021 | AFA-12442-T1 | AFRIMS | Bangkok | June, 1984 | 2.4.0 | 96.9 | - | - |
| ERS447222 | ERR586721 | 13244_5#25 | Ty022 | AFA-12487-T1 | AFRIMS | Bangkok | June, 1984 | 2.4.0 | 77.2 | - | - |
| ERS447223 | ERR586722 | 13244_5#26 | Ty023 | AFA-10558-T1 | AFRIMS | Loi/Northeast | May, 1985 | 2.4.0 | 85.6 | - | - |
| ERS447225 | ERR586723 | 13244_5#27 | Ty025 | AFA-11442-T1 | AFRIMS | Loi/Northeast | May, 1985 | 3.4.0 | 86.4 | - | - |
| ERS447226 | ERR586724 | 13244_5#28 | Ty026 | AFA-11443-T1 | AFRIMS | Loi/Northeast | May, 1985 | 3.4.0 | 88.6 | - | - |
| ERS447227 | ERR586725 | 13244_5#29 | Ty027 | AFA-11444-T1 | AFRIMS | Loi/Northeast | May, 1985 | 2.4.0 | 87.0 | - | - |
| ERS447228 | ERR586802 | 13244_6#12 | Ty028 | AFA-11657-T1 | AFRIMS | Aranyaprathet/Srakaew/East | May, 1986 | 2.1.7 | 101.0 | - | - |
| ERS447230 | ERR586803 | 13244_6#13 | Ty030 | AFA-10552-T1 | AFRIMS | Aranyaprathet/Srakaew/East | May, 1986 | 2.1.7 | 101.0 | - | - |
| ERS447231 | ERR586804 | 13244_6#14 | Ty031 | AFA-10555-T1 | AFRIMS | Aranyaprathet/Srakaew/East | August, 1986 | 3.0.0 | 98.5 | - | - |
| ERS447232 | ERR586805 | 13244_6#15 | Ty032 | AFA-10556-T1 | AFRIMS | Aranyaprathet/Srakaew/East | May, 1986 | 2.1.7 | 99.4 | - | - |
| ERS447233 | ERR586726 | 13244_5#30 | Ty033 | AFA-10557-T1 | AFRIMS | Aranyaprathet/Srakaew/East | January, 1987 | 3.2.1 | 83.8 | - | - |
| ERS447234 | ERR586727 | 13244_5#31 | Ty034 | AFA-10559-T1 | AFRIMS | Aranyaprathet/Srakaew/East | August, 1986 | 2.1.7 | 79.8 | - | - |
| ERS447235 | ERR586728 | 13244_5#32 | Ty036 | AFA-10561-T1 | AFRIMS | Aranyaprathet/Srakaew/East | August, 1986 | 3.2.1 | 83.3 | *aadA1, catA1, sul1, tet(B)* | FIA/HI1 |
| ERS447236 | ERR586806 | 13244_6#16 | Ty037 | AFA-10563-T1 | AFRIMS | Aranyaprathet/Srakaew/East | August, 1986 | 2.1.7 | 89.0 | - | - |
| ERS447237 | ERR586729 | 13244_5#33 | Ty039 | AFA-10565-T1 | AFRIMS | Aranyaprathet/Srakaew/East | July, 1986 | 3.2.1 | 75.9 | - | - |
| ERS447238 | ERR586730 | 13244_5#34 | Ty040 | AFA-11651-T1 | AFRIMS | Aranyaprathet/Srakaew/East | May, 1986 | 2.1.7 | 76.4 | - | - |
| ERS447240 | ERR586807 | 13244_6#17 | Ty042 | AFA-11653-T1 | AFRIMS | Aranyaprathet/Srakaew/East | June, 1986 | 3.0.0 | 91.1 | - | - |
| ERS447241 | ERR586808 | 13244_6#18 | Ty043 | AFA-11654-T1 | AFRIMS | Aranyaprathet/Srakaew/East | June, 1986 | 3.2.1 | 92.0 | - | - |
| ERS447243 | ERR586731 | 13244_5#35 | Ty045 | AFA-11658-T1 | AFRIMS | Aranyaprathet/Srakaew/East | June, 1986 | 2.1.7 | 81.9 | - | - |
| ERS447244 | ERR586732 | 13244_5#36 | Ty046 | AFA-11661-T1 | AFRIMS | Aranyaprathet/Srakaew/East | May, 1986 | 3.2.1 | 80.2 | *aadA1, catA1, sul1, tet(B)* | FIA/HI1 |
| ERS447245 | ERR586809 | 13244_6#19 | Ty047 | AFA-11667-T1 | AFRIMS | Aranyaprathet/Srakaew/East | May, 1986 | 2.1.7 | 91.0 | - | - |
| ERS447246 | ERR586810 | 13244_6#20 | Ty048 | AFA-11668-T1 | AFRIMS | Aranyaprathet/Srakaew/East | April, 1986 | 2.1.7 | 95.2 | - | - |
| ERS447247 | ERR586811 | 13244_6#21 | Ty049 | AFA-11669-T1 | AFRIMS | Aranyaprathet/Srakaew/East | April, 1986 | 3.0.0 | 90.7 | - | - |
| ERS447248 | ERR586812 | 13244_6#22 | Ty050 | AFA-11670-T1 | AFRIMS | Aranyaprathet/Srakaew/East | April, 1986 | 2.1.7 | 95.5 | - | - |
| ERS447249 | ERR586733 | 13244_5#37 | Ty051 | AFA-11671-T1 | AFRIMS | Aranyaprathet/Srakaew/East | June, 1986 | 3.2.1 | 85.1 | *aadA!, catA1, sul1, tet(B)* | FIA/HI1 |
| ERS447250 | ERR586734 | 13244_5#38 | Ty052 | AFA-11672-T1 | AFRIMS | Aranyaprathet/Srakaew/East | June, 1986 | 3.2.1 | 82.2 | *aadA1, catA1, sul1, tet(B)* | FIA/HI1 |
| ERS447251 | ERR586735 | 13244_5#39 | Ty053 | AFA-11673-T1 | AFRIMS | Aranyaprathet/Srakaew/East | April, 1986 | 3.2.1 | 77.3 | - | - |
| ERS447252 | ERR586736 | 13244_5#40 | Ty054 | AFA-10996-T1 | AFRIMS | Aranyaprathet/Srakaew/East | July, 1989 | 3.1.2 | 85.8 | - | - |
| ERS447253 | ERR586737 | 13244_5#41 | Ty055 | AFA-13095-T1 | AFRIMS | Bangkok | May, 1992 | 3.1.2 | 74.7 | - | - |
