# Supplementary material for: Whole Genome Sequence Analysis of Salmonella Typhi Isolated in Thailand before and after the Introduction of a National Immunization Program
Source: PLoS Negl Trop Dis. 2017 Jan 6;11(1):e0005274. doi: 10.1371/journal.pntd.0005274 (PMC5245908; doi:10.1371/journal.pntd.0005274)
Supplement: S2 Table — (DOCX) [file pntd.0005274.s002.docx]

**S2 Table: Global isolate and sequencing details**

| Sample Accession | Sanger ID | Strain | Source | Isolation location | Isolation date | Genotype |
| --- | --- | --- | --- | --- | --- | --- |
| ERR326689 | 9953_5#93 | 9953_5_93_LaoLNT722_2008 | Wong et al 2015 | Laos | 2008 | 3.4.0 |
| ERR326688 | 9953_5#92 | 9953_5_92_LaoLNT72_2007 | Wong et al 2015 | Laos | 2007 | 2.3.4 |
| ERR326687 | 9953_5#91 | 9953_5_91_LaoLNT705_2008 | Wong et al 2015 | Laos | 2008 | 4.1.0 |
| ERR326686 | 9953_5#90 | 9953_5_90_LaoLNT670_2008 | Wong et al 2015 | Laos | 2008 | 3.4.0 |
| ERR326605 | 9953_5#9 | 9953_5_9_IndoA275_2009 | Wong et al 2015 | Indonesia | 2009 | 4.1.0 |
| ERR326685 | 9953_5#89 | 9953_5_89_LaoLNT666_2008 | Wong et al 2015 | Laos | 2008 | 3.4.0 |
| ERR326684 | 9953_5#88 | 9953_5_88_LaoLNT662_2008 | Wong et al 2015 | Laos | 2008 | 2.3.4 |
| ERR326683 | 9953_5#87 | 9953_5_87_LaoLNT659_2008 | Wong et al 2015 | Laos | 2008 | 3.4.0 |
| ERR326682 | 9953_5#86 | 9953_5_86_LaoLNT609_2008 | Wong et al 2015 | Laos | 2008 | 3.4.0 |
| ERR326681 | 9953_5#85 | 9953_5_85_LaoLNT565_2008 | Wong et al 2015 | Laos | 2008 | 3.4.0 |
| ERR326680 | 9953_5#84 | 9953_5_84_LaoLNT375_2008 | Wong et al 2015 | Laos | 2008 | 3.4.0 |
| ERR326679 | 9953_5#83 | 9953_5_83_LaoLNT366_2008 | Wong et al 2015 | Laos | 2008 | 3.4.0 |
| ERR326678 | 9953_5#82 | 9953_5_82_LaoLNT330_2008 | Wong et al 2015 | Laos | 2008 | 2.4.0 |
| ERR326677 | 9953_5#81 | 9953_5_81_LaoLNT279_2008 | Wong et al 2015 | Laos | 2008 | 3.4.0 |
| ERR326676 | 9953_5#80 | 9953_5_80_LaoLNT266_2008 | Wong et al 2015 | Laos | 2008 | 3.4.0 |
| ERR326604 | 9953_5#8 | 9953_5_8_IndoA274_2006 | Wong et al 2015 | Indonesia | 2006 | 3.0.0 |
| ERR326674 | 9953_5#78 | 9953_5_78_LaoLNT1542_2010 | Wong et al 2015 | Laos | 2010 | 3.4.0 |
| ERR326673 | 9953_5#77 | 9953_5_77_LaoLNT1516_2010 | Wong et al 2015 | Laos | 2010 | 3.4.0 |
| ERR326671 | 9953_5#75 | 9953_5_75_LaoLNT1497_2010 | Wong et al 2015 | Laos | 2010 | 3.4.0 |
| ERR326670 | 9953_5#74 | 9953_5_74_LaoLNT1426_2010 | Wong et al 2015 | Laos | 2010 | 3.4.0 |
| ERR326669 | 9953_5#73 | 9953_5_73_LaoLNT1378_2010 | Wong et al 2015 | Laos | 2010 | 3.4.0 |
| ERR326668 | 9953_5#72 | 9953_5_72_LaoLNT1377_2010 | Wong et al 2015 | Laos | 2010 | 2.3.4 |
| ERR326667 | 9953_5#71 | 9953_5_71_LaoLNT1374_2010 | Wong et al 2015 | Laos | 2010 | 3.4.0 |
| ERR326666 | 9953_5#70 | 9953_5_70_LaoLNT1367-3_2010 | Wong et al 2015 | Laos | 2010 | 2.3.4 |
| ERR326603 | 9953_5#7 | 9953_5_7_IndoA273_2009 | Wong et al 2015 | Indonesia | 2009 | 4.1.0 |
| ERR326665 | 9953_5#69 | 9953_5_69_LaoLNT1365_2010 | Wong et al 2015 | Laos | 2010 | 2.3.4 |
| ERR326664 | 9953_5#68 | 9953_5_68_LaoLNT1360_2010 | Wong et al 2015 | Laos | 2010 | 2.4.0 |
| ERR326663 | 9953_5#67 | 9953_5_67_LaoLNT1339_2010 | Wong et al 2015 | Laos | 2010 | 3.4.0 |
| ERR326662 | 9953_5#66 | 9953_5_66_LaoLNT13_2007 | Wong et al 2015 | Laos | 2007 | 3.4.0 |
| ERR326661 | 9953_5#65 | 9953_5_65_LaoLNT12_2007 | Wong et al 2015 | Laos | 2007 | 3.4.0 |
| ERR326660 | 9953_5#64 | 9953_5_64_LaoLNT1197_2009 | Wong et al 2015 | Laos | 2009 | 2.3.4 |
| ERR326659 | 9953_5#63 | 9953_5_63_LaoKM1648_2010 | Wong et al 2015 | Laos | 2010 | 3.4.0 |
| ERR326658 | 9953_5#62 | 9953_5_62_LaoKM1647_2010 | Wong et al 2015 | Laos | 2010 | 3.4.0 |
| ERR326657 | 9953_5#61 | 9953_5_61_LaoKM1646_2010 | Wong et al 2015 | Laos | 2010 | 3.4.0 |
| ERR326656 | 9953_5#60 | 9953_5_60_IndoA399_2009 | Wong et al 2015 | Indonesia | 2009 | 3.1.2 |
| ERR326602 | 9953_5#6 | 9953_5_6_IndoA272_2010 | Wong et al 2015 | Indonesia | 2010 | 4.1.0 |
| ERR326655 | 9953_5#59 | 9953_5_59_IndoA396_2009 | Wong et al 2015 | Indonesia | 2009 | 4.1.0 |
| ERR326654 | 9953_5#58 | 9953_5_58_IndoA395_2009 | Wong et al 2015 | Indonesia | 2009 | 4.1.0 |
| ERR326652 | 9953_5#56 | 9953_5_56_IndoA392_2010 | Wong et al 2015 | Indonesia | 2010 | 4.1.0 |
| ERR326651 | 9953_5#55 | 9953_5_55_IndoA390_2007 | Wong et al 2015 | Indonesia | 2007 | 4.1.0 |
| ERR326649 | 9953_5#53 | 9953_5_53_IndoA385_2012 | Wong et al 2015 | Indonesia | 2012 | 4.1.0 |
| ERR326647 | 9953_5#51 | 9953_5_51_IndoA380_2006 | Wong et al 2015 | Indonesia | 2006 | 3.0.0 |
| ERR326646 | 9953_5#50 | 9953_5_50_IndoA379_2010 | Wong et al 2015 | Indonesia | 2010 | 4.1.0 |
| ERR326601 | 9953_5#5 | 9953_5_5_IndoA271_2009 | Wong et al 2015 | Indonesia | 2009 | 4.1.0 |
| ERR326641 | 9953_5#45 | 9953_5_45_IndoA374_2008 | Wong et al 2015 | Indonesia | 2008 | 4.1.0 |
| ERR326639 | 9953_5#43 | 9953_5_43_IndoA370_2010 | Wong et al 2015 | Indonesia | 2010 | 3.1.2 |
| ERR326638 | 9953_5#42 | 9953_5_42_IndoA369_2008 | Wong et al 2015 | Indonesia | 2008 | 4.1.0 |
| ERR326634 | 9953_5#38 | 9953_5_38_IndoA363_2009 | Wong et al 2015 | Indonesia | 2009 | 3.1.2 |
| ERR326633 | 9953_5#37 | 9953_5_37_IndoA360_2009 | Wong et al 2015 | Indonesia | 2009 | 4.1.0 |
| ERR326632 | 9953_5#36 | 9953_5_36_IndoA359_2010 | Wong et al 2015 | Indonesia | 2010 | 3.1.2 |
| ERR326631 | 9953_5#35 | 9953_5_35_IndoA358_2009 | Wong et al 2015 | Indonesia | 2009 | 4.1.0 |
| ERR326630 | 9953_5#34 | 9953_5_34_IndoA357_2009 | Wong et al 2015 | Indonesia | 2009 | 3.1.2 |
| ERR326627 | 9953_5#31 | 9953_5_31_IndoA354_2011 | Wong et al 2015 | Indonesia | 2011 | 3.0.0 |
| ERR326626 | 9953_5#30 | 9953_5_30_IndoA352_2010 | Wong et al 2015 | Indonesia | 2010 | 3.0.0 |
| ERR326599 | 9953_5#3 | 9953_5_3_IndoA268_2009 | Wong et al 2015 | Indonesia | 2009 | 4.1.0 |
| ERR326622 | 9953_5#26 | 9953_5_26_IndoA344-1st_2010 | Wong et al 2015 | Indonesia | 2010 | 4.1.0 |
| ERR326619 | 9953_5#23 | 9953_5_23_IndoA341_2008 | Wong et al 2015 | Indonesia | 2008 | 3.1.2 |
| ERR326616 | 9953_5#20 | 9953_5_20_IndoA287_2008 | Wong et al 2015 | Indonesia | 2008 | 3.1.2 |
| ERR326614 | 9953_5#18 | 9953_5_18_IndoA285_2010 | Wong et al 2015 | Indonesia | 2010 | 4.1.0 |
| ERR326613 | 9953_5#17 | 9953_5_17_IndoA284_2011 | Wong et al 2015 | Indonesia | 2011 | 3.0.0 |
| ERR326612 | 9953_5#16 | 9953_5_16_IndoA283_2010 | Wong et al 2015 | Indonesia | 2010 | 4.1.0 |
| ERR326611 | 9953_5#15 | 9953_5_15_IndoA282_2010 | Wong et al 2015 | Indonesia | 2010 | 3.1.2 |
| ERR326610 | 9953_5#14 | 9953_5_14_IndoA281_2008 | Wong et al 2015 | Indonesia | 2008 | 3.0.0 |
| ERR326608 | 9953_5#12 | 9953_5_12_IndoA278_2009 | Wong et al 2015 | Indonesia | 2009 | 4.1.0 |
| ERR326607 | 9953_5#11 | 9953_5_11_IndoA277_2009 | Wong et al 2015 | Indonesia | 2009 | 4.1.0 |
| ERR326606 | 9953_5#10 | 9953_5_10_IndoA276_2008 | Wong et al 2015 | Indonesia | 2008 | 4.1.0 |
| ERR319462 | 9870_8#61 | 9870_8_61_Cam1325_2011 | Wong et al 2015 | Cambodia | 2011 | 3.2.1 |
| ERR319418 | 9870_8#17 | 9870_8_17_Cam010587_2009 | Wong et al 2015 | Cambodia | 2009 | 3.2.1 |
| ERR279177 | 9475_6#80 | 9475_6_80_MalA32420_2004 | Wong et al 2015 | Malawi | 2004 | 2.2.0 |
| ERR279169 | 9475_6#72 | 9475_6_72_MalA55865_2009 | Wong et al 2015 | Malawi | 2009 | 2.2.0 |
| ERR279098 | 9475_6#1 | 9475_6_1_Mal1010996_2011 | Wong et al 2015 | Malawi | 2011 | 2.2.0 |
| ERR420428 | 8616_4#52 | 8616_4_52_IndBCR232_2011 | Wong et al 2015 | India | 2011 | 2.0.0 |
| ERR213264 | 8490_6#46 | 8490_6_46_FijMDUST46_1996 | Wong et al 2015 | Fiji | 1996 | 2.0.0 |
| ERR360995 | 10608_2#62 | 10608_2_62_Cam014737_2012 | Wong et al 2015 | Cambodia | 2012 | 4.1.0 |
| ERR360820 | 10607_2#28 | 10607_2_28_MalD55055_2010 | Wong et al 2015 | Malawi | 2010 | 2.2.0 |
| ERR360747 | 10607_1#51 | 10607_1_51_Cam002168_2010 | Wong et al 2015 | Cambodia | 2010 | 3.4.0 |
| ERR360691 | 10593_2#78 | 10593_2_78_LebE01-7006_2001 | Wong et al 2015 | Lebanon | 2001 | 4.1.0 |
| ERR360688 | 10593_2#75 | 10593_2_75_AlgE99-8095_1999 | Wong et al 2015 | Algeria | 1999 | 4.1.0 |
| ERR360673 | 10593_2#60 | 10593_2_60_TurE01-0407_2001 | Wong et al 2015 | Turkey | 2001 | 4.1.0 |
| ERR360664 | 10593_2#51 | 10593_2_51_IndE02-2612_2002 | Wong et al 2015 | India | 2002 | 2.2.0 |
| ERR360618 | 10593_2#5 | 10593_2_5_IndE00-2756_2000 | Wong et al 2015 | India | 2000 | 2.0.0 |
| ERR360661 | 10593_2#48 | 10593_2_48_Vie73-1102_1973 | Wong et al 2015 | Vietnam | 1973 | 4.1.0 |
| ERR360656 | 10593_2#43 | 10593_2_43_MorE00-7878_2000 | Wong et al 2015 | Morocco | 2000 | 2.0.0 |
| ERR360649 | 10593_2#36 | 10593_2_36_Vie72-1907_1972 | Wong et al 2015 | Vietnam | 1972 | 3.4.0 |
| ERR360642 | 10593_2#29 | 10593_2_29_Indo76-1406_1976 | Wong et al 2015 | Indonesia | 1976 | 3.2.1 |
| ERR360637 | 10593_2#24 | 10593_2_24_EgyE99-2862_1999 | Wong et al 2015 | Egypt | 1999 | 2.2.0 |
| ERR360635 | 10593_2#22 | 10593_2_22_MaliE01-1811_2001 | Wong et al 2015 | Mali | 2001 | 2.2.0 |
| ERR360614 | 10593_2#1 | 10593_2_1_MadE00-3459_2000 | Wong et al 2015 | Madagascar | 2000 | 2.2.0 |
| ERR360521 | 10592_2#73 | 10592_2_73_SriLanE01-8716_2001 | Wong et al 2015 | SriLanka | 2001 | 3.0.0 |
| ERR360455 | 10592_2#7 | 10592_2_7_Pak3802_2003 | Wong et al 2015 | Pakistan | 2003 | 2.0.0 |
| ERR360514 | 10592_2#66 | 10592_2_66_MadE96-12081_1996 | Wong et al 2015 | Madagascar | 1996 | 4.1.0 |
| ERR360509 | 10592_2#61 | 10592_2_61_Mad80-2002_1980 | Wong et al 2015 | Madagascar | 1980 | 2.2.0 |
| ERR360503 | 10592_2#55 | 10592_2_55_ArmE98-11555_1998 | Wong et al 2015 | Armenia | 1998 | 2.0.0 |
| ERR360502 | 10592_2#54 | 10592_2_54_MorE00-6657_2000 | Wong et al 2015 | Morocco | 2000 | 4.1.0 |
| ERR360499 | 10592_2#51 | 10592_2_51_MorE99-4879_1999 | Wong et al 2015 | Morocco | 1999 | 2.0.0 |
| ERR360453 | 10592_2#5 | 10592_2_5_Pak17311_2003 | Wong et al 2015 | Pakistan | 2003 | 2.0.0 |
| ERR360491 | 10592_2#43 | 10592_2_43_ComE99-9794_1999 | Wong et al 2015 | Comoros | 1999 | 3.0.0 |
| ERR360487 | 10592_2#39 | 10592_2_39_MorE99-8013_1999 | Wong et al 2015 | Morocco | 1999 | 2.2.0 |
| ERR360466 | 10592_2#18 | 10592_2_18_Pak6289_2003 | Wong et al 2015 | Pakistan | 2003 | 2.0.0 |
| ERR360462 | 10592_2#14 | 10592_2_14_Pak200_2003 | Wong et al 2015 | Pakistan | 2003 | 2.0.0 |
| ERR357825 | 10562_2#70 | 10562_2_70_IndMDUST380_2011 | Wong et al 2015 | India | 2011 | 3.2.1 |
| ERR357823 | 10562_2#68 | 10562_2_68_IndMDUST378_2011 | Wong et al 2015 | India | 2011 | 3.0.0 |
| ERR357761 | 10562_2#6 | 10562_2_6_PhilMDUST134_2011 | Wong et al 2015 | Phillipines | 2011 | 3.0.0 |
| ERR357811 | 10562_2#56 | 10562_2_56_PNGMDUST359_1996 | Wong et al 2015 | PapuaNewGuinea | 1996 | 2.1.7 |
| ERR357810 | 10562_2#55 | 10562_2_55_PNGMDUST357_1994 | Wong et al 2015 | PapuaNewGuinea | 1994 | 2.1.7 |
| ERR357809 | 10562_2#54 | 10562_2_54_PNGMDUST353_1986 | Wong et al 2015 | PapuaNewGuinea | 1986 | 4.1.0 |
| ERR357804 | 10562_2#49 | 10562_2_49_PNGMDUST338_1993 | Wong et al 2015 | PapuaNewGuinea | 1993 | 2.1.7 |
| ERR357802 | 10562_2#47 | 10562_2_47_VanMDUST314_1991 | Wong et al 2015 | Vanuatu | 1991 | 4.1.0 |
| ERR357800 | 10562_2#45 | 10562_2_45_PNGMDUST302_1980 | Wong et al 2015 | PapuaNewGuinea | 1980 | 4.1.0 |
| ERR357798 | 10562_2#43 | 10562_2_43_PNGMDUST275_1992 | Wong et al 2015 | PapuaNewGuinea | 1992 | 2.1.7 |
| ERR357667 | 10561_2#92 | 10561_2_92_Viety1-30_1993 | Wong et al 2015 | Vietnam | 1993 | 3.2.1 |
| ERR357665 | 10561_2#90 | 10561_2_90_Viety2-69_1994 | Wong et al 2015 | Vietnam | 1994 | 4.1.0 |
| ERR357663 | 10561_2#88 | 10561_2_88_Viety2-68_1994 | Wong et al 2015 | Vietnam | 1994 | 3.0.0 |
| ERR357648 | 10561_2#73 | 10561_2_73_Viedtc5_1994 | Wong et al 2015 | Vietnam | 1994 | 3.2.1 |
| ERR357646 | 10561_2#71 | 10561_2_71_Viedtc131_1995 | Wong et al 2015 | Vietnam | 1995 | 3.4.0 |
| ERR357481 | 10541_2#56 | 10541_2_56_PNGMDUST360_1998 | Wong et al 2015 | PapuaNewGuinea | 1998 | 2.1.7 |
| ERR357480 | 10541_2#55 | 10541_2_55_PNGMDUST356_1994 | Wong et al 2015 | PapuaNewGuinea | 1994 | 2.1.7 |
| ERR357478 | 10541_2#53 | 10541_2_53_PNGMDUST345_1985 | Wong et al 2015 | PapuaNewGuinea | 1985 | 2.1.7 |
| ERR357473 | 10541_2#48 | 10541_2_48_FijMDUST334_1984 | Wong et al 2015 | Fiji | 1984 | 3.0.0 |
| ERR357472 | 10541_2#47 | 10541_2_47_PNGMDUST333_1992 | Wong et al 2015 | PapuaNewGuinea | 1992 | 2.1.7 |
| ERR357471 | 10541_2#46 | 10541_2_46_PNGMDUST332_1992 | Wong et al 2015 | PapuaNewGuinea | 1992 | 2.1.7 |
| ERR357470 | 10541_2#45 | 10541_2_45_PNGMDUST329_1992 | Wong et al 2015 | PapuaNewGuinea | 1992 | 2.1.7 |
| ERR357462 | 10541_2#37 | 10541_2_37_VanMDUST315_1991 | Wong et al 2015 | Vanuatu | 1991 | 4.1.0 |
| ERR357456 | 10541_2#31 | 10541_2_31_PNGMDUST305_1990 | Wong et al 2015 | PapuaNewGuinea | 1990 | 2.1.7 |
| ERR357455 | 10541_2#30 | 10541_2_30_PNGMDUST304_1990 | Wong et al 2015 | PapuaNewGuinea | 1990 | 2.1.7 |
| ERR357452 | 10541_2#27 | 10541_2_27_PNGMDUST293_2009 | Wong et al 2015 | PapuaNewGuinea | 2009 | 2.1.7 |
| ERR357451 | 10541_2#26 | 10541_2_26_SamMDUST285_2004 | Wong et al 2015 | Samoa | 2004 | 4.1.0 |
| ERR357450 | 10541_2#25 | 10541_2_25_SamMDUST284_2004 | Wong et al 2015 | Samoa | 2004 | 4.1.0 |
| ERR357449 | 10541_2#24 | 10541_2_24_PNGMDUST281_2002 | Wong et al 2015 | PapuaNewGuinea | 2002 | 2.1.7 |
| ERR357447 | 10541_2#22 | 10541_2_22_PNGMDUST277_2001 | Wong et al 2015 | PapuaNewGuinea | 2001 | 2.1.7 |
| ERR352945 | 10541_2#12 | 10541_2_12_IndoMDUST188_2011 | Wong et al 2015 | Indonesia | 2011 | 3.0.0 |
| ERR352941 | 10541_2#1 | 10541_2_1_PNGMDUST107_2007 | Wong et al 2015 | PapuaNewGuinea | 2007 | 2.1.7 |
| ERR353341 | 10541_1#74 | 10541_1_74_ArgST821_98_1905 | Wong et al 2015 | Argentina | 1905 | 4.1.0 |
| ERR353339 | 10541_1#71 | 10541_1_71_ArgST472_01_1905 | Wong et al 2015 | Argentina | 1905 | 4.1.0 |
| ERR353338 | 10541_1#68 | 10541_1_68_ArgST3090_99_1999 | Wong et al 2015 | Argentina | 1999 | 2.0.0 |
| ERR353334 | 10541_1#62 | 10541_1_62_ArgST1625_88_1988 | Wong et al 2015 | Argentina | 1988 | 4.1.0 |
| ERR353330 | 10541_1#57 | 10541_1_57_ArgST1134_01_2001 | Wong et al 2015 | Argentina | 2001 | 2.0.0 |
| ERR352705 | 10540_1#86 | 10540_1_86_Viety2-138_1994 | Wong et al 2015 | Vietnam | 1994 | 4.1.0 |
| ERR352699 | 10540_1#83 | 10540_1_83_Viety3-199_1997 | Wong et al 2015 | Vietnam | 1997 | 3.2.1 |
| ERR352687 | 10540_1#77 | 10540_1_77_Viety3-213_1997 | Wong et al 2015 | Vietnam | 1997 | 3.2.1 |
| ERR460335 | 10540_1#4 | 10540_1_4_IndoE03-4983_2003 | Wong et al 2015 | Indonesia | 2003 | 3.1.2 |
| ERR352511 | 10493_1#86 | 10493_1_86_UnkMDUST415_2012 | Wong et al 2015 | Unknown | 2012 | 3.0.0 |
| ERR352503 | 10493_1#78 | 10493_1_78_PNGMDUST358_1996 | Wong et al 2015 | PapuaNewGuinea | 1996 | 2.1.7 |
| ERR352500 | 10493_1#75 | 10493_1_75_PNGMDUST348_1985 | Wong et al 2015 | PapuaNewGuinea | 1985 | 2.1.7 |
| ERR352497 | 10493_1#72 | 10493_1_72_PNGMDUST331_1992 | Wong et al 2015 | PapuaNewGuinea | 1992 | 2.1.7 |
| ERR352496 | 10493_1#71 | 10493_1_71_PNGMDUST330_1992 | Wong et al 2015 | PapuaNewGuinea | 1992 | 2.1.7 |
| ERR352493 | 10493_1#68 | 10493_1_68_PNGMDUST313_1994 | Wong et al 2015 | PapuaNewGuinea | 1994 | 2.1.7 |
| ERR352492 | 10493_1#67 | 10493_1_67_IndMDUST301_1988 | Wong et al 2015 | India | 1988 | 2.1.7 |
| ERR352491 | 10493_1#66 | 10493_1_66_PNGMDUST300_1998 | Wong et al 2015 | PapuaNewGuinea | 1998 | 2.1.7 |
| ERR352490 | 10493_1#65 | 10493_1_65_PNGMDUST299_1998 | Wong et al 2015 | PapuaNewGuinea | 1998 | 2.1.7 |
| ERR352489 | 10493_1#64 | 10493_1_64_PNGMDUST298_1998 | Wong et al 2015 | PapuaNewGuinea | 1998 | 2.1.7 |
| ERR352487 | 10493_1#62 | 10493_1_62_PNGMDUST296_2010 | Wong et al 2015 | PapuaNewGuinea | 2010 | 2.1.7 |
| ERR352483 | 10493_1#58 | 10493_1_58_PNGMDUST288_2007 | Wong et al 2015 | PapuaNewGuinea | 2007 | 2.1.7 |
| ERR352482 | 10493_1#57 | 10493_1_57_PNGMDUST287_2007 | Wong et al 2015 | PapuaNewGuinea | 2007 | 2.1.7 |
| ERR352478 | 10493_1#53 | 10493_1_53_PNGMDUST279_2002 | Wong et al 2015 | PapuaNewGuinea | 2002 | 2.1.7 |
| ERR352477 | 10493_1#52 | 10493_1_52_PNGMDUST278_2001 | Wong et al 2015 | PapuaNewGuinea | 2001 | 2.1.7 |
| ERR352476 | 10493_1#51 | 10493_1_51_PNGMDUST276_1994 | Wong et al 2015 | PapuaNewGuinea | 1994 | 2.1.7 |
| ERR352469 | 10493_1#44 | 10493_1_44_VieMDUST235_2010 | Wong et al 2015 | Vietnam | 2010 | 3.2.1 |
| ERR352465 | 10493_1#40 | 10493_1_40_IndMDUST215_2011 | Wong et al 2015 | India | 2011 | 3.0.0 |
| ERR352462 | 10493_1#37 | 10493_1_37_BanMDUST205_2010 | Wong et al 2015 | Bangladesh | 2010 | 2.0.0 |
| ERR352461 | 10493_1#36 | 10493_1_36_BanMDUST204_2010 | Wong et al 2015 | Bangladesh | 2010 | 2.0.0 |
| ERR352460 | 10493_1#35 | 10493_1_35_BanMDUST203_2010 | Wong et al 2015 | Bangladesh | 2010 | 2.0.0 |
| ERR352459 | 10493_1#34 | 10493_1_34_BanMDUST202_2010 | Wong et al 2015 | Bangladesh | 2010 | 2.0.0 |
| ERR352427 | 10493_1#2 | 10493_1_2_PNGMDUST109_2007 | Wong et al 2015 | PapuaNewGuinea | 2007 | 2.1.7 |
| ERR352441 | 10493_1#16 | 10493_1_16_IndoMDUST150_2012 | Wong et al 2015 | Indonesia | 2012 | 3.0.0 |
| ERR352435 | 10493_1#10 | 10493_1_10_PhilMDUST129_2011 | Wong et al 2015 | Phillipines | 2011 | 3.0.0 |
| ERR352337 | 10492_1#84 | 10492_1_84_UnkMDUST411_2011 | Wong et al 2015 | Unknown | 2011 | 4.1.0 |
| ERR352331 | 10492_1#78 | 10492_1_78_IndoMDUST397_2011 | Wong et al 2015 | Indonesia | 2011 | 3.0.0 |
| ERR352328 | 10492_1#75 | 10492_1_75_IndMDUST391_2011 | Wong et al 2015 | India | 2011 | 2.2.0 |
| ERR352260 | 10492_1#7 | 10492_1_7_MalayMDUST130_2011 | Wong et al 2015 | Malaysia | 2011 | 3.0.0 |
| ERR352322 | 10492_1#69 | 10492_1_69_PNGMDUST364_1999 | Wong et al 2015 | PapuaNewGuinea | 1999 | 2.1.7 |
| ERR352321 | 10492_1#68 | 10492_1_68_PNGMDUST363_1998 | Wong et al 2015 | PapuaNewGuinea | 1998 | 2.1.7 |
| ERR352320 | 10492_1#67 | 10492_1_67_PNGMDUST362_1998 | Wong et al 2015 | PapuaNewGuinea | 1998 | 2.1.7 |
| ERR352319 | 10492_1#66 | 10492_1_66_PNGMDUST361_1998 | Wong et al 2015 | PapuaNewGuinea | 1998 | 2.1.7 |
| ERR352318 | 10492_1#65 | 10492_1_65_PNGMDUST355_1994 | Wong et al 2015 | PapuaNewGuinea | 1994 | 2.1.7 |
| ERR352314 | 10492_1#61 | 10492_1_61_PNGMDUST328_1992 | Wong et al 2015 | PapuaNewGuinea | 1992 | 2.1.7 |
| ERR352309 | 10492_1#56 | 10492_1_56_PNGMDUST274_1996 | Wong et al 2015 | PapuaNewGuinea | 1996 | 2.1.7 |
| ERR352301 | 10492_1#48 | 10492_1_48_PNGMDUST256_2010 | Wong et al 2015 | PapuaNewGuinea | 2010 | 2.1.7 |
| ERR352300 | 10492_1#47 | 10492_1_47_PNGMDUST255_2010 | Wong et al 2015 | PapuaNewGuinea | 2010 | 2.1.7 |
| ERR352299 | 10492_1#46 | 10492_1_46_IndoMDUST253_2010 | Wong et al 2015 | Indonesia | 2010 | 3.1.2 |
| ERR352288 | 10492_1#35 | 10492_1_35_PNGMDUST229_2010 | Wong et al 2015 | PapuaNewGuinea | 2010 | 2.1.7 |
| ERR352287 | 10492_1#34 | 10492_1_34_PNGMDUST226_2010 | Wong et al 2015 | PapuaNewGuinea | 2010 | 2.1.7 |
| ERR352285 | 10492_1#32 | 10492_1_32_IndMDUST217_2011 | Wong et al 2015 | India | 2011 | 3.2.1 |
| ERR352282 | 10492_1#29 | 10492_1_29_IndoMDUST206_2010 | Wong et al 2015 | Indonesia | 2010 | 3.0.0 |
| ERR352255 | 10492_1#2 | 10492_1_2_PNGMDUST115_2009 | Wong et al 2015 | PapuaNewGuinea | 2009 | 2.1.7 |
| ERR352269 | 10492_1#16 | 10492_1_16_ChinMDUST154_2011 | Wong et al 2015 | China | 2011 | 2.3.4 |
| ERR349604 | 10426_1#83 | 10426_1_83_Viety2-115_1994 | Wong et al 2015 | Vietnam | 1994 | 4.1.0 |
| ERR349575 | 10426_1#54 | 10426_1_54_Vieas257_1999 | Wong et al 2015 | Vietnam | 1999 | 3.2.1 |
| ERR349559 | 10426_1#37 | 10426_1_37_Viety2-166_1994 | Wong et al 2015 | Vietnam | 1994 | 2.2.0 |
| ERR349537 | 10426_1#15 | 10426_1_15_Viedn110_1996 | Wong et al 2015 | Vietnam | 1996 | 3.4.0 |
| ERR349426 | 10425_1#96 | 10425_1_96_Viety2-75_1994 | Wong et al 2015 | Vietnam | 1994 | 3.2.1 |
| ERR349423 | 10425_1#93 | 10425_1_93_Viety2-86_1994 | Wong et al 2015 | Vietnam | 1994 | 3.2.1 |
| ERR349415 | 10425_1#85 | 10425_1_85_Viety2-99_1994 | Wong et al 2015 | Vietnam | 1994 | 3.2.1 |
| ERR349412 | 10425_1#82 | 10425_1_82_Viety2-111_1994 | Wong et al 2015 | Vietnam | 1994 | 2.1.7 |
| ERR349411 | 10425_1#81 | 10425_1_81_Viety2-98_1994 | Wong et al 2015 | Vietnam | 1994 | 3.2.1 |
| ERR349406 | 10425_1#76 | 10425_1_76_Viety2-80_1994 | Wong et al 2015 | Vietnam | 1994 | 3.4.0 |
| ERR349402 | 10425_1#72 | 10425_1_72_Viety2-93_1994 | Wong et al 2015 | Vietnam | 1994 | 4.1.0 |
| ERR349400 | 10425_1#70 | 10425_1_70_Viety2-32_1993 | Wong et al 2015 | Vietnam | 1993 | 3.2.1 |
| ERR349397 | 10425_1#67 | 10425_1_67_Viety2-91_1994 | Wong et al 2015 | Vietnam | 1994 | 3.2.1 |
| ERR349384 | 10425_1#54 | 10425_1_54_Viety3-214_1997 | Wong et al 2015 | Vietnam | 1997 | 4.1.0 |
| ERR349376 | 10425_1#46 | 10425_1_46_Vieipt57_1997 | Wong et al 2015 | Vietnam | 1997 | 2.3.4 |
| ERR349370 | 10425_1#40 | 10425_1_40_Viect1-7_1993 | Wong et al 2015 | Vietnam | 1993 | 3.2.1 |
| ERR349362 | 10425_1#32 | 10425_1_32_Viedtc111_1994 | Wong et al 2015 | Vietnam | 1994 | 4.1.0 |
| ERR349351 | 10425_1#21 | 10425_1_21_Viedtc116_1995 | Wong et al 2015 | Vietnam | 1995 | 3.4.0 |
| ERR349343 | 10425_1#13 | 10425_1_13_RusBRD948_1996 | Wong et al 2015 | Russia | 1996 | 4.1.0 |
| ERR349340 | 10425_1#10 | 10425_1_10_UnkM223_1939 | Wong et al 2015 | Unknown | 1939 | 2.1.7 |
| ERR352253 | 10396_8#20 | 10396_8_20_LaoUI3257_2003 | Wong et al 2015 | Laos | 2003 | 3.2.1 |
| ERR343338 | 10349_1#90 | 10349_1_90_Indo404ty_1983 | Wong et al 2015 | Indonesia | 1983 | 3.1.2 |
| ERR343332 | 10349_1#84 | 10349_1_84_RusTy2_1916 | Wong et al 2015 | Russia | 1916 | 4.1.0 |
| ERR343329 | 10349_1#81 | 10349_1_81_IndH12ESR02737_2012 | Wong et al 2015 | India | 2012 | 2.4.0 |
| ERR343328 | 10349_1#80 | 10349_1_80_PhilH12ESR00755-001A_2012 | Wong et al 2015 | Phillipines | 2012 | 3.0.0 |
| ERR343256 | 10349_1#8 | 10349_1_8_Indo02732_2002 | Wong et al 2015 | Indonesia | 2002 | 3.1.2 |
| ERR343326 | 10349_1#78 | 10349_1_78_Ind12959_2012 | Wong et al 2015 | India | 2012 | 3.1.2 |
| ERR343324 | 10349_1#76 | 10349_1_76_Ind12590_2012 | Wong et al 2015 | India | 2012 | 4.1.0 |
| ERR343318 | 10349_1#70 | 10349_1_70_Samer114070_2011 | Wong et al 2015 | SouthAmerica | 2011 | 2.2.0 |
| ERR343317 | 10349_1#69 | 10349_1_69_Nep114000_2011 | Wong et al 2015 | Nepal | 2011 | 2.2.0 |
| ERR343291 | 10349_1#43 | 10349_1_43_Indo091092_2009 | Wong et al 2015 | Indonesia | 2009 | 3.1.2 |
| ERR343289 | 10349_1#41 | 10349_1_41_Ind08758_2008 | Wong et al 2015 | India | 2008 | 2.1.7 |
| ERR343287 | 10349_1#39 | 10349_1_39_Chin084170_2008 | Wong et al 2015 | China | 2008 | 3.1.2 |
| ERR343265 | 10349_1#17 | 10349_1_17_Sam041932_2004 | Wong et al 2015 | Samoa | 2004 | 4.1.0 |
| ERR343263 | 10349_1#15 | 10349_1_15_Ind041419_2004 | Wong et al 2015 | India | 2004 | 4.1.0 |
| ERR340765 | 10209_5#9 | 10209_5_9_Cam007073_2007 | Wong et al 2015 | Cambodia | 2007 | 3.2.1 |
| ERR340816 | 10209_5#62 | 10209_5_62_LaoUI54_2000 | Wong et al 2015 | Laos | 2000 | 4.1.0 |
| ERR340813 | 10209_5#59 | 10209_5_59_LaoUI3313_2003 | Wong et al 2015 | Laos | 2003 | 3.4.0 |
| ERR340811 | 10209_5#57 | 10209_5_57_LaoUI3260_2003 | Wong et al 2015 | Laos | 2003 | 3.2.1 |
| ERR340810 | 10209_5#54 | 10209_5_54_LaoUI3151_2002 | Wong et al 2015 | Laos | 2002 | 2.3.4 |
| ERR340808 | 10209_5#52 | 10209_5_52_LaoUI3146_2002 | Wong et al 2015 | Laos | 2002 | 2.3.4 |
| ERR340805 | 10209_5#49 | 10209_5_49_LaoUI2679_2002 | Wong et al 2015 | Laos | 2002 | 3.2.1 |
| ERR340804 | 10209_5#48 | 10209_5_48_LaoUI2658_2002 | Wong et al 2015 | Laos | 2002 | 3.4.0 |
| ERR340803 | 10209_5#47 | 10209_5_47_LaoUI2591_2002 | Wong et al 2015 | Laos | 2002 | 3.4.0 |
| ERR340801 | 10209_5#45 | 10209_5_45_LaoUI2351_2002 | Wong et al 2015 | Laos | 2002 | 3.2.1 |
| ERR340798 | 10209_5#42 | 10209_5_42_LaoUI2152_2002 | Wong et al 2015 | Laos | 2002 | 4.1.0 |
| ERR340793 | 10209_5#37 | 10209_5_37_LaoUI2006_2002 | Wong et al 2015 | Laos | 2002 | 3.2.1 |
| ERR340788 | 10209_5#32 | 10209_5_32_LaoUI1954-2_3_2002 | Wong et al 2015 | Laos | 2002 | 3.2.1 |
| ERR340786 | 10209_5#30 | 10209_5_30_LaoUI1236_2001 | Wong et al 2015 | Laos | 2001 | 3.2.1 |
| ERR340785 | 10209_5#29 | 10209_5_29_LaoUI1203_2001 | Wong et al 2015 | Laos | 2001 | 3.2.1 |
| ERR340782 | 10209_5#26 | 10209_5_26_LaoLNT358_2000 | Wong et al 2015 | Laos | 2000 | 3.4.0 |
| ERR340781 | 10209_5#25 | 10209_5_25_LaoLNT26_2001 | Wong et al 2015 | Laos | 2001 | 3.4.0 |
| ERR340780 | 10209_5#24 | 10209_5_24_LaoLNT22_2001 | Wong et al 2015 | Laos | 2001 | 3.4.0 |
| ERR340779 | 10209_5#23 | 10209_5_23_LaoLNT1148_2001 | Wong et al 2015 | Laos | 2001 | 3.4.0 |
| ERR340778 | 10209_5#22 | 10209_5_22_LaoLNT1108_2001 | Wong et al 2015 | Laos | 2001 | 3.4.0 |
| ERR340777 | 10209_5#21 | 10209_5_21_LaoLNT08_2001 | Wong et al 2015 | Laos | 2001 | 3.4.0 |
| ERR340769 | 10209_5#13 | 10209_5_13_Cam100239_2007 | Wong et al 2015 | Cambodia | 2007 | 3.2.1 |
| ERR338129 | 10071_8#60 | 10071_8_60_Sam0060_2012 | Wong et al 2015 | Samoa | 2012 | 4.1.0 |
| ERR338128 | 10071_8#59 | 10071_8_59_Sam0059_2012 | Wong et al 2015 | Samoa | 2012 | 4.1.0 |
| ERR338099 | 10071_8#30 | 10071_8_30_Sam0030_2012 | Wong et al 2015 | Samoa | 2012 | 4.1.0 |
| ERR338069 | 10071_3#93 | 10071_3_93_IndoA137_2008 | Wong et al 2015 | Indonesia | 2008 | 4.1.0 |
| ERR338068 | 10071_3#92 | 10071_3_92_indoA136_2010 | Wong et al 2015 | Indonesia | 2010 | 3.1.2 |
| ERR338067 | 10071_3#91 | 10071_3_91_IndoA135_2004 | Wong et al 2015 | Indonesia | 2004 | 3.1.2 |
| ERR338065 | 10071_3#89 | 10071_3_89_IndoA132_2011 | Wong et al 2015 | Indonesia | 2011 | 3.1.2 |
| ERR338064 | 10071_3#88 | 10071_3_88_IndoA131_2004 | Wong et al 2015 | Indonesia | 2004 | 3.0.0 |
| ERR338062 | 10071_3#86 | 10071_3_86_IndoA128_2008 | Wong et al 2015 | Indonesia | 2008 | 3.1.2 |
| ERR338060 | 10071_3#84 | 10071_3_84_IndoA125_2005 | Wong et al 2015 | Indonesia | 2005 | 3.1.2 |
| ERR338058 | 10071_3#82 | 10071_3_82_IndoA122_2005 | Wong et al 2015 | Indonesia | 2005 | 3.1.2 |
| ERR338051 | 10071_3#75 | 10071_3_75_IndoA105_2012 | Wong et al 2015 | Indonesia | 2012 | 3.1.2 |
| ERR338047 | 10071_3#71 | 10071_3_71_Safr634660_2012 | Wong et al 2015 | S.Africa | 2012 | 2.4.0 |
| ERR338044 | 10071_3#68 | 10071_3_68_Safr400746_2009 | Wong et al 2015 | S.Africa | 2009 | 2.4.0 |
| ERR338029 | 10071_3#53 | 10071_3_53_Safr410378_2009 | Wong et al 2015 | S.Africa | 2009 | 2.4.0 |
| ERR338025 | 10071_3#49 | 10071_3_49_Safr516981_2010 | Wong et al 2015 | S.Africa | 2010 | 2.4.0 |
| ERR338020 | 10071_3#44 | 10071_3_44_Safr1026656_2004 | Wong et al 2015 | S.Africa | 2004 | 2.4.0 |
| ERR338019 | 10071_3#43 | 10071_3_43_Safr1650362_2005 | Wong et al 2015 | S.Africa | 2005 | 2.4.0 |
| ERR338013 | 10071_3#37 | 10071_3_37_Safr257401_2008 | Wong et al 2015 | S.Africa | 2008 | 2.4.0 |
| ERR338011 | 10071_3#35 | 10071_3_35_Safr238675_2008 | Wong et al 2015 | S.Africa | 2008 | 2.4.0 |
| ERR338009 | 10071_3#33 | 10071_3_33_Safr225555_2007 | Wong et al 2015 | S.Africa | 2007 | 2.4.0 |
| ERR338005 | 10071_3#29 | 10071_3_29_Safr1647624_2005 | Wong et al 2015 | S.Africa | 2005 | 2.4.0 |
| ERR338001 | 10071_3#25 | 10071_3_25_Safr175806_2006 | Wong et al 2015 | S.Africa | 2006 | 2.4.0 |
| ERR331306 | 10060_6#9 | 10060_6_9_LaoSV179_2009 | Wong et al 2015 | Laos | 2009 | 3.2.1 |
| ERR331381 | 10060_6#84 | 10060_6_84_Tan62717_2006 | Wong et al 2015 | Tanzania | 2006 | 2.2.0 |
| ERR331305 | 10060_6#8 | 10060_6_8_LaoSV171_2009 | Wong et al 2015 | Laos | 2009 | 3.2.1 |
| ERR331370 | 10060_6#73 | 10060_6_73_Tan61025_2006 | Wong et al 2015 | Tanzania | 2006 | 2.2.0 |
| ERR331304 | 10060_6#7 | 10060_6_7_LaoSV170_2009 | Wong et al 2015 | Laos | 2009 | 3.2.1 |
| ERR331365 | 10060_6#68 | 10060_6_68_LaoXN211_2010 | Wong et al 2015 | Laos | 2010 | 3.4.0 |
| ERR331364 | 10060_6#67 | 10060_6_67_LaoXK1684_2010 | Wong et al 2015 | Laos | 2010 | 3.2.1 |
| ERR331363 | 10060_6#66 | 10060_6_66_LaoXK1682_2010 | Wong et al 2015 | Laos | 2010 | 3.2.1 |
| ERR331361 | 10060_6#64 | 10060_6_64_LaoUX309_2009 | Wong et al 2015 | Laos | 2009 | 3.4.0 |
| ERR331360 | 10060_6#63 | 10060_6_63_LaoUX305_2009 | Wong et al 2015 | Laos | 2009 | 3.4.0 |
| ERR331359 | 10060_6#62 | 10060_6_62_LaoUX229_2009 | Wong et al 2015 | Laos | 2009 | 3.4.0 |
| ERR331358 | 10060_6#61 | 10060_6_61_LaoUI5685-2_2004 | Wong et al 2015 | Laos | 2004 | 4.1.0 |
| ERR331357 | 10060_6#60 | 10060_6_60_LaoUI5669_2004 | Wong et al 2015 | Laos | 2004 | 4.1.0 |
| ERR331303 | 10060_6#6 | 10060_6_6_LaoSV108_2008 | Wong et al 2015 | Laos | 2008 | 3.2.1 |
| ERR331356 | 10060_6#59 | 10060_6_59_LaoUI5557_2004 | Wong et al 2015 | Laos | 2004 | 3.4.0 |
| ERR331355 | 10060_6#58 | 10060_6_58_LaoUI5446_2004 | Wong et al 2015 | Laos | 2004 | 3.4.0 |
| ERR331354 | 10060_6#57 | 10060_6_57_LaoUI5312_2004 | Wong et al 2015 | Laos | 2004 | 3.2.1 |
| ERR331353 | 10060_6#56 | 10060_6_56_LaoUI5275_2004 | Wong et al 2015 | Laos | 2004 | 3.2.1 |
| ERR331350 | 10060_6#53 | 10060_6_53_LaoUI5026_2004 | Wong et al 2015 | Laos | 2004 | 3.4.0 |
| ERR331349 | 10060_6#52 | 10060_6_52_LaoUI4940_2004 | Wong et al 2015 | Laos | 2004 | 4.1.0 |
| ERR331302 | 10060_6#5 | 10060_6_5_LaoST716_2008 | Wong et al 2015 | Laos | 2008 | 3.4.0 |
| ERR331345 | 10060_6#48 | 10060_6_48_LaoUI3930_2003 | Wong et al 2015 | Laos | 2003 | 3.4.0 |
| ERR331343 | 10060_6#46 | 10060_6_46_LaoUI3862_2003 | Wong et al 2015 | Laos | 2003 | 3.4.0 |
| ERR331342 | 10060_6#45 | 10060_6_45_LaoUI3816_2003 | Wong et al 2015 | Laos | 2003 | 3.4.0 |
| ERR331341 | 10060_6#44 | 10060_6_44_LaoUI3753_2003 | Wong et al 2015 | Laos | 2003 | 3.2.1 |
| ERR331340 | 10060_6#43 | 10060_6_43_LaoUI3744_2003 | Wong et al 2015 | Laos | 2003 | 3.4.0 |
| ERR331339 | 10060_6#42 | 10060_6_42_LaoUI3608_2003 | Wong et al 2015 | Laos | 2003 | 2.2.0 |
| ERR331337 | 10060_6#40 | 10060_6_40_LaoUI3564_2003 | Wong et al 2015 | Laos | 2003 | 4.1.0 |
| ERR331334 | 10060_6#37 | 10060_6_37_LaoUI3446_2003 | Wong et al 2015 | Laos | 2003 | 3.0.0 |
| ERR331332 | 10060_6#35 | 10060_6_35_LaoUI17614_2010 | Wong et al 2015 | Laos | 2010 | 3.2.1 |
| ERR331331 | 10060_6#34 | 10060_6_34_LaoUI17187_2010 | Wong et al 2015 | Laos | 2010 | 4.1.0 |
| ERR331329 | 10060_6#32 | 10060_6_32_LaoUI16161_2010 | Wong et al 2015 | Laos | 2010 | 3.4.0 |
| ERR331300 | 10060_6#3 | 10060_6_3_LaoLNT899_2009 | Wong et al 2015 | Laos | 2009 | 3.4.0 |
| ERR331326 | 10060_6#29 | 10060_6_29_LaoUI14191_3_2009 | Wong et al 2015 | Laos | 2009 | 3.4.0 |
| ERR331324 | 10060_6#27 | 10060_6_27_LaoUI13797_2009 | Wong et al 2015 | Laos | 2009 | 3.2.1 |
| ERR331323 | 10060_6#26 | 10060_6_26_LaoUI13599_2009 | Wong et al 2015 | Laos | 2009 | 3.2.1 |
| ERR331322 | 10060_6#25 | 10060_6_25_LaoUI13529-2_2009 | Wong et al 2015 | Laos | 2009 | 3.4.0 |
| ERR331321 | 10060_6#24 | 10060_6_24_LaoUI12162_2008 | Wong et al 2015 | Laos | 2008 | 3.2.1 |
| ERR331320 | 10060_6#23 | 10060_6_23_LaoUI11955_2008 | Wong et al 2015 | Laos | 2008 | 3.4.0 |
| ERR331319 | 10060_6#22 | 10060_6_22_LaoUI11562_2008 | Wong et al 2015 | Laos | 2008 | 4.1.0 |
| ERR331318 | 10060_6#21 | 10060_6_21_LaoUI11483_2008 | Wong et al 2015 | Laos | 2008 | 3.4.0 |
| ERR331316 | 10060_6#19 | 10060_6_19_LaoUI10006_2007 | Wong et al 2015 | Laos | 2007 | 2.3.4 |
| ERR331311 | 10060_6#14 | 10060_6_14_LaoSV431_2009 | Wong et al 2015 | Laos | 2009 | 3.2.1 |
| ERR331308 | 10060_6#11 | 10060_6_11_LaoSV211_2009 | Wong et al 2015 | Laos | 2009 | 3.2.1 |
| ERR331307 | 10060_6#10 | 10060_6_10_LaoSV200_2009 | Wong et al 2015 | Laos | 2009 | 3.4.0 |
| ERR331298 | 10060_6#1 | 10060_6_1_LaoLNT757_2009 | Wong et al 2015 | Laos | 2009 | 2.3.4 |
| ERR331257 | 10060_5#54 | 10060_5_54_IndoA266_2010 | Wong et al 2015 | Indonesia | 2010 | 4.1.0 |
| ERR331256 | 10060_5#53 | 10060_5_53_IndoA265_2010 | Wong et al 2015 | Indonesia | 2010 | 4.1.0 |
| ERR331254 | 10060_5#51 | 10060_5_51_IndoA263_2009 | Wong et al 2015 | Indonesia | 2009 | 4.1.0 |
| ERR331251 | 10060_5#48 | 10060_5_48_IndoA259_2010 | Wong et al 2015 | Indonesia | 2010 | 4.1.0 |
| ERR331250 | 10060_5#47 | 10060_5_47_IndoA258_2009 | Wong et al 2015 | Indonesia | 2009 | 4.1.0 |
| ERR331249 | 10060_5#46 | 10060_5_46_IndoA257_2010 | Wong et al 2015 | Indonesia | 2010 | 3.0.0 |
| ERR331248 | 10060_5#45 | 10060_5_45_IndoA256_2010 | Wong et al 2015 | Indonesia | 2010 | 3.0.0 |
| ERR331247 | 10060_5#44 | 10060_5_44_IndoA255_2011 | Wong et al 2015 | Indonesia | 2011 | 4.1.0 |
| ERR331244 | 10060_5#41 | 10060_5_41_IndoA252_2011 | Wong et al 2015 | Indonesia | 2011 | 4.1.0 |
| ERR331243 | 10060_5#40 | 10060_5_40_IndoA250_2009 | Wong et al 2015 | Indonesia | 2009 | 4.1.0 |
| ERR331242 | 10060_5#39 | 10060_5_39_IndoA249_2010 | Wong et al 2015 | Indonesia | 2010 | 3.1.2 |
| ERR331241 | 10060_5#38 | 10060_5_38_IndoA248_2009 | Wong et al 2015 | Indonesia | 2009 | 3.0.0 |
| ERR331240 | 10060_5#37 | 10060_5_37_IndoA247_2010 | Wong et al 2015 | Indonesia | 2010 | 4.1.0 |
| ERR331239 | 10060_5#36 | 10060_5_36_IndoA246_2010 | Wong et al 2015 | Indonesia | 2010 | 4.1.0 |
| ERR331238 | 10060_5#35 | 10060_5_35_IndoA245_2009 | Wong et al 2015 | Indonesia | 2009 | 4.1.0 |
| ERR331237 | 10060_5#34 | 10060_5_34_IndoA244_2011 | Wong et al 2015 | Indonesia | 2011 | 3.0.0 |
| ERR331236 | 10060_5#33 | 10060_5_33_IndoA243_2011 | Wong et al 2015 | Indonesia | 2011 | 4.1.0 |
| ERR331234 | 10060_5#31 | 10060_5_31_IndoA240_2010 | Wong et al 2015 | Indonesia | 2010 | 4.1.0 |
| ERR331233 | 10060_5#30 | 10060_5_30_IndoA239_2009 | Wong et al 2015 | Indonesia | 2009 | 3.1.2 |
| ERR331232 | 10060_5#29 | 10060_5_29_IndoA238_2009 | Wong et al 2015 | Indonesia | 2009 | 4.1.0 |
| ERR331231 | 10060_5#28 | 10060_5_28_IndoA237_2010 | Wong et al 2015 | Indonesia | 2010 | 3.0.0 |
| ERR331230 | 10060_5#27 | 10060_5_27_IndoA236_2009 | Wong et al 2015 | Indonesia | 2009 | 4.1.0 |
| ERR331229 | 10060_5#26 | 10060_5_26_IndoA235_2009 | Wong et al 2015 | Indonesia | 2009 | 3.0.0 |
| ERR331227 | 10060_5#24 | 10060_5_24_IndoA180_2009 | Wong et al 2015 | Indonesia | 2009 | 4.1.0 |
| ERR331226 | 10060_5#23 | 10060_5_23_IndoA178_2009 | Wong et al 2015 | Indonesia | 2009 | 3.1.2 |
| ERR331220 | 10060_5#17 | 10060_5_17_IndoA158_2009 | Wong et al 2015 | Indonesia | 2009 | 3.1.2 |
| ERR331219 | 10060_5#16 | 10060_5_16_IndoA156_2007 | Wong et al 2015 | Indonesia | 2007 | 3.0.0 |
| ERR331215 | 10060_5#12 | 10060_5_12_IndoA150_2006 | Wong et al 2015 | Indonesia | 2006 | 3.0.0 |
